# The Impact of Variant Histology in Patients with Urothelial Carcinoma Treated with Radical Cystectomy: Can We Predict the Presence of Variant Histology?

**DOI:** 10.3390/curroncol30100638

**Published:** 2023-09-27

**Authors:** Nebojsa Prijovic, Miodrag Acimovic, Veljko Santric, Branko Stankovic, Predrag Nikic, Ivan Vukovic, Milan Radovanovic, Luka Kovacevic, Petar Nale, Uros Babic

**Affiliations:** 1 Clinic of Urology, University Clinical Center of Serbia, Resavska Str. 51, 11000 Belgrade, Serbia; nebojsa.prijovic@yahoo.com (N.P.); miodrag.acimovic@med.bg.ac.rs (M.A.); veljkosantric@yahoo.com (V.S.); bstank@gmail.com (B.S.); nicksha@gmail.com (P.N.); ivanvukovic.urolog@gmail.com (I.V.); milan_950@hotmail.com (M.R.); kovac.luka13@gmail.com (L.K.); petarnale@gmail.com (P.N.); 2 Faculty of Medicine, University of Belgrade, Dr Subotica Str. 8, 11000 Belgrade, Serbia

**Keywords:** bladder cancer, urothelial carcinoma, variant histology, radical cystectomy, neutrophils, predictor

## Abstract

Considering the divergent biological behaviors of certain histological subtypes of urothelial carcinoma, it would be of great importance to examine the impact of variant histology and to predict its presence in patients with bladder cancer. A single-center cohort study included 459 patients who underwent radical cystectomy for urothelial carcinoma between 2017 and 2021. Patients were followed up with until July 2022. We compared clinical, laboratory, and histopathologic characteristics and the overall survival between patients with pure urothelial carcinoma and variant histologies. Our results showed that the patients with variant histology were older and preoperatively more frequently had hydronephrosis and higher values of leukocytes and neutrophils. Also, we found a significant association between variant histology and an advanced stage of tumor disease, the presence of lymphovascular invasion, positive surgical margins, and metastases in surgically resected lymph nodes. The number of neutrophils was identified as an independent preoperative predictor of the presence of variant histology after a radical cystectomy. The overall survival of the patients with variant histology was significantly lower compared to the patients with pure urothelial carcinoma. According to our results, the presence of variant histology represents a more aggressive form of the disease. Preoperative neutrophil counts may indicate the presence of variant histology of urothelial carcinoma in patients with bladder cancer.

## 1. Introduction

Bladder cancer (BC) is the 10th most common malignancy worldwide, with male predominance both in incidence and in mortality rates [1]. In the developed world, histopathologically, between 90% and 95% of BC cases are urothelial carcinoma [2]. Depending on the invasion of the muscle layer of the bladder, BC is classified as non-muscle-invasive BC (NMIBC) and muscle-invasive BC (MIBC) [3]. The standard treatment for patients with MIBC, as well as selected patients with high-risk NMIBC, is radical cystectomy [4,5]. However, despite radical surgical treatment, less than half of patients with MIBC live for longer than 5 years [6]. Therefore, the identification of the prognostic markers of the disease is of great importance. The so-far identified histopathological characteristics of BC related to the disease prognosis are the stage of the tumor disease, the presence of metastases in the lymph nodes, and the presence of lymphovascular invasion [7,8]. Currently, extensive research is being conducted to examine the impact of histopathological heterogeneity of urothelial carcinoma on the course and prognosis of BC. In pathology reports, pure urothelial carcinoma is found in about 75% of cases, while variant histologies of urothelial carcinoma, which represent aberrant differentiation, are seen in 25% of cases [9]. However, it is assumed that up to 50% of variant histologies are not recognized by pathologists, especially if the pathologist is not an expert in urogenital pathology [10,11]. Furthermore, variant histologies are more often found in samples that are obtained after radical cystectomy than via transurethral resection of bladder tumor (TURBT) [10]. Since the divergent biological behavior of certain histological subtypes of urothelial carcinoma and different responses to standard treatment modalities have been observed [12], it would be of great importance to predict the presence of variant histologies before surgery and identify it in surgical samples after surgery in a timely manner. 

In light of the current trends in BC trials, the aim of our study is to examine the association of variant histology of urothelial carcinoma in patients who were treated with radical cystectomy with the clinical and histopathological characteristics, as well as with overall survival (OS). Also, the aim of this research is to identify predictors of the presence of variant histology among the preoperative characteristics of patients who were treated with radical cystectomy.

## 2. Materials and Methods

### 2.1. The Cohort and Baseline Characteristics

This was a retrospective single-center study, and the data used for the purposes of this study refer to the database containing all patients who underwent radical cystectomy for primary bladder cancer during the period from 1 January 2017 to 31 December 2021 at the Urology Clinic of the University Clinical Center of Serbia in Belgrade. The detailed methodology has already been described in a study that was previously published [13]. 

The key eligibility criterion for this study was a pathohistologically confirmed diagnosis of urothelial BC after radical cystectomy. This study did not include patients with non-urothelial BC after radical cystectomy and patients who underwent salvage cystectomy. Moreover, this study did not include patients with hematological diseases and immunodeficiency conditions characterized by disturbances in the blood count. Blood samples were taken immediately after admission and were analyzed in the laboratory of the Center for Medical Biochemistry of the University Clinical Center of Serbia in Belgrade. The histopathological diagnoses were determined by an experienced uropathologist. The BC type and variant histology of urothelial carcinoma were determined according to the current World Health Organization classification [14]. The tumor stage was determined according to the current TNM classification of bladder cancer [15]. The data cutoff for this analysis was 30 June 2022, and patients were followed up for a period of at least 6 months after enrollment, e.g., after radical cystectomy. The primary outcome was overall survival (OS), and it was calculated from the date of the radical cystectomy to death from any cause or the data cut-off date. Patients who were lost to follow-up and those who were still alive at the end of analysis were censored in statistical analysis. Data on survival were obtained during routine hospital visits and/or by contacting patients by phone.

### 2.2. Statistical Analysis

We used the descriptive and analytical statistics methods to process the obtained data. We analyzed the differences in characteristics between patients with pure urothelial carcinoma and patients with variant histologies. The existence of a difference between variables that were subject to normal distribution was examined using Student’s T-test for independent samples, while the Mann–Whitney U test was used for variables that were not subject to normal distribution. The differences in the frequencies of variables were analyzed using the chi-squared test or Fisher’s exact probability test. We constructed Kalpan–Meier survival curves for the patients with pure urothelial carcinoma and variant histology. We analyzed the difference in overall survival between these two groups of patients using the log rank test. In order to identify independent predictors of the presence of variant histology, preoperative characteristics with a statistically significant difference were analyzed using the binary logistic regression analysis in a multivariate model. We considered a value of *p* < 0.05 to be statistically significant. For data analysis, we used the SPSS software, version 20 for Windows.

## 3. Results

The study included 459 patients who underwent radical cystectomy for urothelial BC. The median age was 67.0 years (interquartile range (IQR) 62.0–72.0 years). Of the total number of patients, 364 (79.3%) were male patients, and 95 (20.7%) were female. Pure urothelial carcinoma was observed in 369 (80.4%) patients, while the presence of variant histologies was found in 90 (19.6%) patients. Considering the tumor stage, pTa and pTis were present in 12 (2.6%) patients, pT1 was present in 37 patients (8.1%), pT2 was present in 158 patients (34.4%), pT3 was present in 143 patients (31.2%), and pT4 stage was present in 109 (23.7%) patients. Lymphovascular invasion was observed in 321 (70.7%) patients. Surgical margins were positive in 67 (14.6%) patients. Lymph node metastases were identified in 76 (23.1%) patients who underwent pelvic lymphadenectomy. The detailed demographic, clinical, and histopathological characteristics are presented in Table 1.

Table 2 shows the prevalence of specific types of histological variants of urothelial carcinoma, with a note that it was possible to detect more than one histological variant in the same sample after radical cystectomy. The most common histological variant was the presence of squamous differentiation, which was found in 46 (10.0%) patients.

The values of laboratory parameters are presented in Table 3. The average hemoglobin value was 121.8 ± 24.7 g/L, the number of leukocytes was 8.2 ± 3.3 × 10^9^/L, and the number of platelets was 265.3 ± 92.1 × 10^9^/L. The average number of neutrophils was 5.4 ± 3.1 × 10^9^/L.

Table 4 shows a comparison of the clinical, laboratory, and histopathological characteristics among patients with pure urothelial carcinoma and variant histologies. The patients with variant histology were significantly older (68.1 ± 8.3 vs. 66.0 ± 7.9) compared to the patients with pure urothelial carcinoma (*p* = 0.027). We observed a significantly higher frequency of preoperative hydronephrosis in the patients with variant histology (43.3%) in comparison with the patients with pure urothelial carcinoma (32.0%) (*p* = 0.042). In the group with variant histologies, there was a significantly higher representation of patients with a higher stage of the primary tumor—the pT3 (38.9% vs. 29.3%) and pT4 stages (31.1% vs. 22.0%) (*p* = 0.002). Lymphovascular invasion was observed significantly more often in the patients with variant histology (86.5%) compared to the patients with pure urothelial carcinoma (66.8%) (*p* < 0.001). Also, the surgical margins were more often positive in the patients with variant histology (23.3% vs. 12.5%) (*p* = 0.009). Lymph node metastases were observed more often in the patients with variant histology (42.2% vs. 18.5%) (*p* < 0.001). Higher values of the number of leukocytes (8.8 ± 3.6 vs. 8.0 ± 3.2) and neutrophils (6.1 ± 3.4 vs. 5.2 ± 3.0) were observed in the patients with variant histology (*p* = 0.044 and *p* = 0.008, respectively).

Table 5 shows the results of binary logistic regression in a multivariate model. When we analyzed the parameters that differed significantly in the patients with pure urothelial carcinoma and variant histologies, the number of neutrophils was noted as an independent predictor of the presence of variant histology (*p* = 0.048).

**Figure 1:**
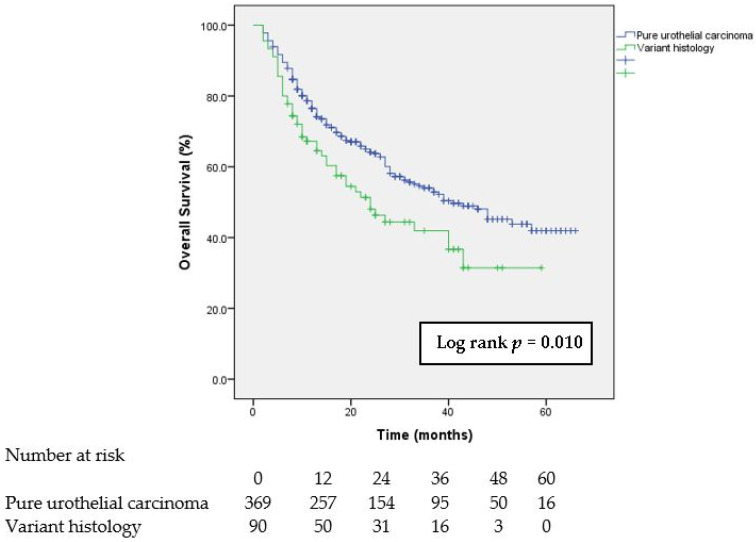
Kaplan–Meier estimate of overall survival for patients with pure urothelial carcinoma and variant histologies treated with radical cystectomy.

At the end of the analysis, 251 (54.7%) patients were alive, while 208 (45.3%) patients died. The OS was calculated from the date of the radical cystectomy, and the median follow-up time was 18 months (range of 2–66 months). The median OS values in the groups of patients with variant histology of urothelial carcinoma and pure urothelial carcinoma were 24.0 months (95% CI, 15.35–32.65) and 41.0 months (95% CI, 29.77–52.23), respectively. Figure 1 shows the Kaplan–Meier estimate of the OS for the patients with pure urothelial carcinoma and variant histologies treated with radical cystectomy. Our analysis showed the significant impact of variant histology on the OS. After radical cystectomy, the patients with variant histology had a significantly lower OS compared to the patients with pure urothelial carcinoma (log-rank *p* = 0.010) 

## 4. Discussion

In the developed world, urothelial carcinoma is the most common type of BC, with a prevalence of over 90% [2]. Variant histologies of urothelial carcinoma, which represent aberrant differentiation, can be seen in about 25–33% of cases [9,16]. The presence of squamous and/or glandular differentiation is detected the most often [17], followed by micropapillary, nested, plasmacytoid, and other types [18] in variant histologies of urothelial carcinoma. Recently, growing evidence highlighted the importance of variant histologies of urothelial carcinoma as prognostic and predictive factors in patients with MIBC [12,19]. It was shown that the use of neoadjuvant chemotherapy does not provide benefits in all variants of urothelial carcinoma [19,20]. Since these patients are often excluded from clinical studies, currently, there is a considerable lack of strong treatment recommendations in clinical practice guidelines for patients with variant histologies of urothelial carcinoma [9].

In our study, we found the presence of variant histology of urothelial carcinoma in 19.6% of patients who underwent a radical cystectomy. According to the available data in the literature, the appearance of variant histology in urothelial carcinoma is 25–33%, and it is found more often in specimens obtained via radical cystectomy than via the TURBT [17]. It is assumed that up to 50% of variant histologies observed after radical cystectomy were not found in TURBT specimens [10]. Reasons such as an irregular representation of variant histology in the entire tumor, the pathologist having a lack of expertise in urogenital pathology, as well as the presence of artifacts after tumor resection may all explain the dissimilarity in the detection of variant histology in urothelial carcinoma [11,21,22]. Therefore, in this study, we did not examine the presence or absence of variant histology in TURBT specimens that were obtained before radical cystectomy. 

When analyzing the demographic and clinical characteristics of our patients, we noticed that the patients with variant histologies were significantly older than the patients with pure urothelial carcinoma. Furthermore, a significantly higher presence of preoperative hydronephrosis was found in the patients with confirmed variant histology in our study. Until now, numerous studies showed the association of preoperative hydronephrosis with an advanced stage of BC [23,24,25] and with even worse survival in these patients [26]. In this study, we found a significantly higher prevalence of the pT3 and pT4 stages of BC in patients with variant histologies of urothelial carcinoma. The findings obtained in our study are consistent with the previously reported results of other studies that variant histology of urothelial carcinoma is associated with a higher grade and more advanced stage of BC [27,28]. To illustrate, the study by Deuker et al. showed a higher TNM stage in patients with variant histologies compared to pure urothelial carcinoma [29]. We did not notice a gender difference between patients with pure urothelial carcinoma and variant histology in our study, although the data from the literature indicate a more advanced stage and higher cancer-specific mortality among women with variant histology [30].

Due to this biological aggressiveness, the presence of variant histology is considered a high-risk disease in all major guidelines for urothelial bladder carcinoma [31]. However, strong recommendations in terms of the use of neoadjuvant chemotherapy or early radical cystectomy in patients with variant histology of urothelial bladder carcinoma are still missing [32].

When observing the association with other histopathological characteristics of BC, we found a higher frequency of lymphovascular invasion (LVI) in patients with variant histology. It is widely accepted that LVI in patients with MIBC indicates a more aggressive disease, and it is considered a significant predictive factor of patient survival after radical cystectomy [8]. Thus, the higher prevalence of LVI in patients with variant histology of urothelial carcinoma usually means a more aggressive form of the disease. Furthermore, in patients with variant histology, we observed a higher frequency of positive surgical margins. In contrast to our findings, La Croce et al. did not show that variant histology was associated with positive surgical margins after radical cystectomy, but in their study, only specimens obtained via TURBT were analyzed [33]. However, the importance of the surgical margins status after a radical cystectomy is still controversial, with conflicting results reported so far [34,35]. Hence, the results of the meta-analysis from 2016 indicate an association of positive surgical margins with worse OS in patients who were treated with radical cystectomy [36]. Seemingly, a higher frequency of positive surgical margins in patients with variant histology suggests a more aggressive biological behavior and the presence of locally advanced disease.

The lymph node status after radical cystectomy represents one of the most significant prognostic factors in these patients [7]. It is well recognized that conventional imaging modalities are limited when detecting metastases in lymph nodes that are not enlarged [37]. Therefore, the identification of potential biomarkers that could facilitate the detection of micrometastases in clinically non-enlarged lymph nodes would be of great importance. In this study, we found that in patients where pelvic lymphadenectomy was performed during radical cystectomy, metastases in lymph nodes were significantly more frequently detected in patients with variant histology of urothelial carcinoma. Our results are consistent with the findings reported by Deuker et al., who found an association of variant histology with a higher TNM stage [29]. Also, in accordance with our findings are the results of La Croce et al., who found that the presence of variant histology was an independent predictor of extravesical disease [33]. Overall, our study showed the association of variant histology with all proven histopathological predictors of poor survival after radical cystectomy. Therefore, our results indicate more aggressive behavior of urothelial carcinoma with variant histology compared to pure urothelial carcinoma. 

It is known that the 5-year survival of patients after a radical cystectomy is less than 50% [6]. Currently, investigators put a lot of effort into the search for prognostic and predictive biomarkers in urothelial carcinoma. The impact of variant histology on the survival of patients with BC, as well as determining the strong recommendations in guidelines for the treatment of these patients, has become the focus of research. 

In this study, we found a significantly lower OS of patients with variant histology compared to patients with pure urothelial carcinoma. This should not be surprising considering that in our study, variant histology was associated with all of the known risk factors for poor OS after radical cystectomy, e.g., a higher stage of tumor disease, the presence of lymphovascular invasion, and positive lymph nodes. The data from the literature on the impact of variant histology on the OS of patients with BC are still controversial, especially due to the different biological behaviors of certain histological subtypes. When considering the two most common variant histologies of urothelial carcinoma—squamous and glandular divergent differentiation—earlier studies have indicated a worse OS for patients with these histopathological types [38,39]. However, the findings of recent studies do not indicate a difference in the OS of patients with these histological subtypes compared to pure urothelial carcinoma [28,40]. Yet, the data on the OS of patients with the micropapillary variant remain controversial [19]. A study by Yue et al. highlighted small cell variant histology as a predictor of worse survival after a radical cystectomy [39]. Bearing in mind the observed differences in the behaviors of variant histologies and pure urothelial carcinoma, one of the goals of our study was to identify the potential predictors of the presence of variant histology in samples that were obtained after a radical cystectomy. Therefore, we analyzed, using multivariate binary logistic regression, the preoperative characteristics that were significantly different in the group of patients with variant histology and in the group with pure urothelial carcinoma. Among the examined factors that differed significantly, e.g., age, preoperative hydronephrosis, number of leukocytes, and number of neutrophils, we identified the number of neutrophils as an independent predictor of the presence of variant histology after radical cystectomy. Accumulating evidence on the interrelation between inflammation and tumor behavior led to the acceptance that currently, inflammation is considered the “seventh sign” of cancer [41]. Therefore, the roles of inflammation and neutrophils in various diseases attract special attention from researchers. Existing knowledge indicates the association of neutrophils and their markers with a worse prognosis in patients with different types of cancer [42]. To illustrate, the study by Yiang et al. identified an elevated level of neutrophils as an independent predictor of the OS after a radical thymoma surgery [43]. The first insight into the mechanism linking the impact of neutrophils to tumorigenesis refers to the influence of reactive oxygen species (ROS) released by leukocytes on DNA damage in the pathogenesis of lung cancer [44]. Furthermore, the role of neutrophils in tumor progression has been explained as well, i.e., their involvement in breaking down the extracellular matrix and promoting neovascularization [45]. Findings from an in vitro study suggest the role of neutrophils in the cellular invasion of BC by modulating androgen receptor (AR)/MMP 13 signals [46]. Moreover, the role of neutrophils in suppressing the anti-tumor activity of other immune cells has been described, which additionally points to the pro-tumor effect of neutrophils [47]. When considering BC, numerous studies have shown the influence of inflammatory parameters on the histopathological characteristics of BC, where the examined indexes were directly proportional to the number of neutrophils in the peripheral blood [13,48,49]. Li DX et al. showed that higher neutrophil-to-lymphocyte ratio levels were associated with a worse OS in patients with variant histology of urothelial carcinoma [50]. Given that higher neutrophil levels create a favorable environment for local tumor progression, our finding that higher neutrophil levels were associated with the presence of variant histology is consistent with the other findings of our study, which indicate a more aggressive behavior of variant histologies of urothelial carcinoma. To the best of our knowledge, there are no published studies that have examined the association of neutrophil count values with the presence of variant histology of urothelial carcinoma.

Our study had several limitations. First, it was a retrospective study, with all limitations marked by its observational nature. Second, it was a single-center study, which can explain the relatively small number of patients that were included. Also, in our study, we compared patients with pure urothelial carcinoma and patients with variant histology, not taking into account the subtypes of histological variants due to the relatively small number of patients with variant histology.

## 5. Conclusions

Our study showed a multiple association of variant histology of urothelial carcinoma with clinical and histopathological characteristics in patients with BC. Patients with variant histology who are treated with a radical cystectomy more often have an advanced stage of tumor disease, the presence of lymphovascular invasion, positive surgical margins, and metastases in surgically resected lymph nodes. Patients with variant histology are older, and preoperatively more frequently have hydronephrosis and higher values of leukocytes and neutrophils. The number of neutrophils was found to be an independent predictor of the presence of variant histology after radical cystectomy. Finally, our study showed a significantly lower OS for patients with variant histology, which, together with other identified associations, signifies a more aggressive form of the disease compared to pure urothelial carcinoma.

Given the fact that readily available preoperative characteristics can potentially be associated with the presence of variant histology, a prospective study with a larger number of patients should be conducted in the future to examine the predictive significance of preoperative clinical and laboratory characteristics, including other inflammatory markers. Also, a prospective study with a larger number of patients would be of great importance to examine the prognostic significance of variant histology in urothelial carcinoma.

## Figures and Tables

**Table 1:** Demographic, clinical, and histopathological characteristics of patients with urothelial carcinoma treated with radical cystectomy.

Total	459
Age (years), median (IQR)	67.0 (62.0–72.0)
Gender, *n* (%)	
Male	364 (79.3)
Female	95 (20.7)
Body mass index (kg/m^2^), median (IQR)	25.7 (23.6–27.9)
Tobacco smoking, *n* (%)	
No	147 (32.0)
Current/former smoker	312 (68.0)
Alcohol consumption, *n* (%)	
No	381 (83.0)
Yes	78 (17.0)
ASA score, *n* (%)	
1 + 2	325 (70.8)
3 + 4	134 (29.2)
ECOG performance status, *n* (%)	
0	185 (40.3)
≥1	274 (59.7)
Preoperative hydronephrosis, *n* (%)	
No	302 (65.8)
Yes	157 (34.2)
Subtypes of urothelial carcinoma, *n* (%)	
Pure urothelial carcinoma	369 (80.4)
Variant histology	90 (19.6)
pT stage, *n*(%)	
pTa + pTis	12 (2.6)
pT1	37 (8.1)
pT2	158 (34.4)
pT3	143 (31.2)
pT4	109 (23.7)
Lymphovascular invasion, *n* (%)	
Absent	133 (29.3)
Present	321 (70.7)
Surgical margins, *n* (%)	
Negative	392 (85.4)
Positive	67 (14.6)
Lymph nodes, *n* (%)	
Negative	253 (76.9)
Positive	76 (23.1)

IQR, interquartile range; ASA, American Society of Anesthesiologists; ECOG, Eastern Cooperative Oncology Group.

**Table 2:** Types and prevalence of variant histology of urothelial carcinoma after radical cystectomy.

Variant Histology	*N* (%) *
Squamous differentiation	46 (10.0)
Nested variant	15 (3.3)
Micropapillary UC	13 (2.8)
Glandular differentiation	9 (2.0)
Lymphoepithelioma-like UC	2 (0.4)
Sarcomatoid UC	8 (1.7)
Plasmocytoid UC	1 (0.2)
Neoroendocrine differentiation	1 (0.2)

* It was possible to detect more than one histology variant in the same specimen.

**Table 3:** Values of laboratory parameters of patients with urothelial carcinoma treated with radical cystectomy.

Parameter	Mean ± SD
Hemoglobin (g/L)	121.8 ± 24.7
Leukocytes (×10^9^/L)	8.2 ± 3.3
Neutrophils (×10^9^/L)	5.4 ± 3.1
Lymphocytes (×10^9^/L)	1.9 ± 0.7
Platelets (×10^9^/L)	265.3 ± 92.1
Total protein (g/L)	68.0 ± 6.5
Albumin (g/L)	39.5 ± 4.6

SD, standard deviation.

**Table 4:** Comparison of demographic, clinical, histopathological, and laboratory characteristics between patients with pure urothelial carcinoma and variant histologies.

Parameter	Pure Urothelial Carcinoma	Variant Histology	*p* Value
Age (years), mean ± SD	66.0 ± 7.9	68.1 ± 8.3	0.027 ^a^
Gender, *n* (%)			
Male	293 (79.4)	71 (78.9)	0.914 ^b^
Female	76 (20.6)	19 (21.1)	
BMI (kg/m^2^) mean ± SD	25.8 ± 3.6	26.2 ± 4.3	0.334 ^a^
Tobacco smoking, *n* (%)			
No	115 (31.2)	32 (35.6)	0.451 ^b^
Current/former smoker	254 (68.8)	58 (64.4)	
Alcohol consumption, *n* (%)			
No	302 (81.8)	79 (87.8)	0.179 ^b^
Yes	67 (18.2)	11 (12.2)	
ASA score, *n* (%)			
1 + 2	261 (70.7)	64 (71.1)	0.943 ^b^
3 + 4	108 (29.3)	26 (28.9)	
ECOG performance status, *n* (%)			
0	149 (40.4)	36 (40.0)	0.948 ^b^
≥1	220 (59.6)	54 (60.0)	
Preoperative hydronephrosis, *n* (%)			
No	251 (68.0)	51 (56.7)	0.042 ^b^
Yes	118 (32.0)	39 (43.3)	
pT stage, *n* (%)			
pTa + pTis	12 (3.3)	0 (0.0)	0.002 ^c^
pT1	36 (9.8)	1 (1.1)	
pT2	132 (35.8)	26 (28.9)	
pT3	108 (29.3)	35 (38.9)	
pT4	81 (22.0)	28 (31.1)	
Lymphovascular invasion, *n* (%)			
Absent	121 (33.2)	12 (13.5)	<0.001 ^b^
Present	244 (66.8)	77 (86.5)	
Surgical margins, *n* (%)			
Negative	323 (87.5)	69 (76.7)	0.009 ^b^
Positive	46 (12.5)	21 (23.3)	
Lymph nodes, *n* (%)			
Negative	216 (81.5)	37 (57.8)	<0.001 ^b^
Positive	49 (18.5)	27 (42.2)	
Hemoglobin (g/L), mean ± SD	122.0 ± 24.9	120.9 ± 24.2	0.717 ^a^
Leukocytes (×10^9^/L), mean ± SD	8.0 ± 3.2	8.8 ± 3.6	0.044 ^d^
Neutrophils (×10^9^/L), mean ± SD	5.2 ± 3.0	6.1 ± 3.4	0.008 ^d^
Lymphocytes (×10^9^/L), mean ± SD	1.9 ± 0.7	1.7 ± 0.6	0.088 ^a^
Platelets (×10^9^/L), mean ± SD	264.1 ± 92.1	269.9 ± 92.3	0.594 ^a^
Total protein (g/L), mean ± SD	67.9 ± 6.4	68.2 ± 6.9	0.628 ^a^
Albumin (g/L), mean ± SD	39.6 ± 4.6	39.2 ± 4.6	0.440 ^a^

^a^ Student’s *t*-test; ^b^ χ2 test; ^c^ Fisher’s exact test; ^d^ Mann–Whitney U test. SD, standard deviation; BMI, Body Mass Index; ASA, American Society of Anesthesiologists; ECOG, Eastern Cooperative Oncology Group.

**Table 5:** Binary logistic analysis of preoperative predictors of the presence of variant histology in patients with urothelial carcinoma treated with radical cystectomy (multivariate model).

Parameter	OR	95% CI	*p* Value
Age	1.031	1.000–1.062	0.051
Preoperative hydronephrosis	1.408	0.861–2.307	0.174
Leukocytes	0.791	0.591–1.058	0.114
Neutrophils	1.367	1.003–1.864	0.048

OR, Odds Ratio; CI, Confidence Interval.

## Data Availability

The datasets used and analyzed during the current study are available from the corresponding authors upon reasonable request.
